# Knotting and unknotting proteins in the chaperonin cage: Effects of the excluded volume

**DOI:** 10.1371/journal.pone.0176744

**Published:** 2017-05-10

**Authors:** Szymon Niewieczerzal, Joanna I. Sulkowska

**Affiliations:** 1 Centre of New Technologies, University of Warsaw, Banacha 2c, 02-097 Warsaw, Poland; 2 Department of Chemistry, University of Warsaw, Pasteura 1, 02-093 Warsaw, Poland; University of Michigan, UNITED STATES

## Abstract

Molecular dynamics simulations are used to explore the effects of chaperonin-like cages on knotted proteins with very low sequence similarity, different depths of a knot but with a similar fold, and the same type of topology. The investigated proteins are VirC2, DndE and MJ0366 with two depths of a knot. A comprehensive picture how encapsulation influences folding rates is provided based on the analysis of different cage sizes and temperature conditions. Neither of these two effects with regard to knotted proteins has been studied by means of molecular dynamics simulations with coarse-grained structure-based models before. We show that encapsulation in a chaperonin is sufficient to self-tie and untie small knotted proteins (VirC2, DndE), for which the equilibrium process is not accessible in the bulk solvent. Furthermore, we find that encapsulation reduces backtracking that arises from the destabilisation of nucleation sites, smoothing the free energy landscape. However, this effect can also be coupled with temperature rise. Encapsulation facilitates knotting at the early stage of folding and can enhance an alternative folding route. Comparison to unknotted proteins with the same fold shows directly how encapsulation influences the free energy landscape. In addition, we find that as the size of the cage decreases, folding times increase almost exponentially in a certain range of cage sizes, in accordance with confinement theory and experimental data for unknotted proteins.

## Introduction

The majority of small globular proteins can fold spontaneously in the cellular environment. However, to ensure folding of complex proteins and prevent them from aggregation, in each of the domains of life so-called chaperone networks emerged [1]. The most universal molecular chaperones are chaperonins. They form a specialised nano-compartment for single protein molecules to allow folding in isolation [2–4]. Recent data suggest that the chaperonin may also actively rescue proteins from kinetic folding traps, thereby accelerating their folding rates [5, 6]. Knotted bacterial proteins [7] represent a class of proteins which could significantly benefit from chaperonins. The folding landscape of those proteins is complex and full of hidden traps due to the necessity of tying a knot on the backbone. Our review of more than 200 chaperonin-dependent proteins tested in [8] shows that indeed, the folding of knotted proteins is supported by chaperonins and some knotted bacterial proteins are stringently chaperonin-dependent. However, it appears that the fact that these proteins are knotted was not realized by the authors of [8]. Proteins which do not take the advantage of chaperonin machinery have to adopt the native structure in a highly crowded cellular environment. Different levels of crowding can be also mimicked by keeping the investigated protein in confinement of varying size. Finding an explanation how the confined space, being a result of either chaperonin confinement or crowded solution, affects knotting, is our goal in this work. Understanding the role of confinement may lead in particular to explanation of how knotted proteins could survive evolutional pressure and whether chaperonins play a role in the structural evolution of knotted and unknotted proteins with similar fold and function.

It is also noted that the folding mechanism of knotted proteins is still not well understood theoretically. Currently, around 1.4% of all structures deposited in the RCSB database are knotted or slipknotted according to KnotProt [7]. Knotted proteins are found in all branches of life, and they are either enzymes or DNA binding proteins [9]. Proteins with the simplest knot (trefoil or 3_1_) are most common in PDB (90% in the non-redundant set of proteins); however, only for two of such proteins an artificially [10] designed knotted protein and the smallest knotted protein MJ0366 (with one knot tail of varying length) determination of the free energy landscape was successful by means of different coarse-grained models [11–13] and all-atom simulations [14, 15]. Even though these two proteins have different folds, the general folding mechanism is similar as a knot is always made at the last step. Depending on the length of a threaded tail, the knot is made either by the so-called slipknot motif, or by plugging one of the ends through a previously formed twisted loop, and its formation corresponds to the rate limiting step. Those routes were observed in all-atom simulations [14, 15]. Our survey across the KnotProt database has shown that a similar fold and the same topology as MJ0366 are present in two other proteins, VirC2 (also with the RHH fold) and the newly deposited DndE with an unclassified fold. However, we also found that even though the knot in VirC2 and DndE is deeper only by 2 and 5 amino acids, respectively, compared to MJ0366, their thermodynamic analysis is not possible without acceleration (contrary to that of MJ0366).

Furthermore, for longer proteins with significantly deeper knots (at least 30 amino acids from both termini), for example those from the SPOUT family, only a few self-tying events were observed in unbiased structure based models (SBM) [16]. This number increases with the introduction of non-native contacts [17]. Independently of the applied model, knotting is the rate limiting step. In vivo experiments have shown that these proteins can self-tie slowly [18, 19]. This process can be speeded up significantly by the chaperonin, which in the first approximation can be modelled by a cage introducing an excluded volume effect. However, the exact role of chaperonin is unknown. On the other hand, indirect influence of the chaperonin is observed for other knotted proteins, which are members of the UCHL family [20], and which can self-tie. Thus the current study shows that the energy landscape of knotted proteins is minimally frustrated, but efficient folding in contrast to folding in the natural environment is not observed theoretically.

It is also well known that the knotting probability of a polymer-like chain significantly grows once they are squeezed, e.g. inside channels, slits, cavities, or upon crowding [21]. The latter case boosts the incidence of physical knots by more than an order of magnitude [22].

To sum up, the folding of knotted proteins is difficult to model theoretically. However, confined space increases chances of protein folding, as well as chances of knotting of polymer-like chains. As in a cell proteins are confined, we may suspect that such an environment may be the key element to effectively tie and untie knotted proteins. This raises many questions, with our study focusing on the following ones: How does the encapsulation influence the folding of knotted proteins; is knot formation still a rate-limiting step? Is the excluded volume effect sufficient to speed up folding? To what extent are knotted proteins dependent on a specific chaperone mechanism, and is such dependence linked to a knot type and depth? Can cellular crowding contribute to the folding of knotted proteins? To our knowledge, so far these questions have not been addressed theoretically (via molecular dynamics simulations).

The chaperonin structure and reaction cycle have been extensively investigated [2, 5, 6, 23, 24]. How encapsulation in a chaperonin facilitates folding is still an open question, because it is very difficult to follow such process with current experimental techniques. In general passive and active mechanisms have been proposed [2, 23]. The passive mechanism assumes that a just excluded volume can prevent the formation of reversible aggregates and thus accelerate spontaneous folding. The active mechanism is postulated in two different models. One model takes into account multiple cycles of protein binding to chaperone which are correlated with conformational changes of the cage. This forces binding and disrupting of any incorrect contacts that may be present in proteins and thus frees them from kinetic traps [24]. Under this assumption acceleration in folding rates arises from more chances to escape from kinetic traps, due to multiple cycles of binding-releasing by chaperonin. The second active model suggests that higher folding rates arise from the elimination of local energy minima that would otherwise serve as kinetic traps for a protein [2, 6]. It is assumed here that chaperonin sufficiently smoothens the energy landscape just in one circle of binding [2]. A few experimental results, such as for bacterial Rubisco (50 kDa) [2] or (41 kDa) maltose-binding protein (DM-MBP) [23], confirm the second active model. Mechanism of chaperonin cycles and their influence on protein folding was also explored theoretically using SBM. In [25], authors considered various chaperonin models with varying interaction strength between the cavity and hydrophobic amino acids of substrate protein, as well as the size of the cavity, mimicking in this way the full chaperonin cycle. These altering conditions with properly adjusted cycle times lead to increase in the native state yield, compared with less complex cavity models. Note that the effect of encapsulation was investigated before for globular proteins with trivial topology by means of structure-based models, which elucidated how it affects their thermal stability and folding mechanism [26–29].

In this article we study the influence of chaperonin-like cavity, as well as the significant reduction of accessible space which imitates confined environment, on the shape of the free energy landscape of knotted proteins. We construct a simple nano-cage [27] and analyse how encapsulation affects thermodynamics and kinetics of knotted proteins with two folds: RHH motif (proteins MJ0366, MJ0366_CC, and VirC2) and DndE protein with an unclassified fold. These proteins have the same topology; however, with different depths of knots and low sequence similarity. We also compare our results to those for an unknotted protein with the RHH motif belonging to a large family of bacterial transcriptional regulators. We show that encapsulation in the chaperonin is enough to self-tie and untie knotted proteins, for which the equilibrium process is not accessible in the bulk solvent. We find that encapsulation smoothens the free energy landscape and eliminates kinetic traps by reducing backtracking that arises from the destabilisation of nucleation sites. Moreover, encapsulation facilitates knotting at the early stage of folding and can enhance an alternative folding route.

## Materials and methods

### Proteins

We study five proteins, four with a trefoil knot and one unknotted. We consider two structures of MJ0366 from Methanocaldococcus jannaschii (pdb code: 2efv), the smallest knotted protein known to date: one 82-residue long which is available in PDB and a structure with a complete C-terminal end, denoted as MJ0366 and MJ0366_CC, respectively. The additional five residues which were not crystallised (E, G, E, R, A) were reconstructed as an extended helix using NAMD. MJ0366 has a ribbon-helix-helix fold known as the RHH fold, a motif characteristic for proteins binding double-stranded DNA.

**Fig 1:**
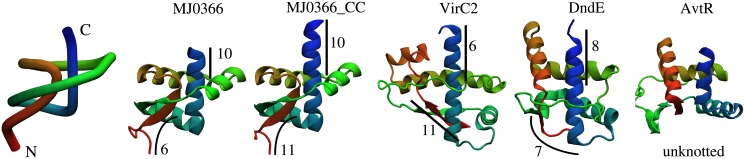
Structures of the investigated proteins: MJ0366 (residue 6-87, pdb code 2efv), MJ0366_CC (residue 6-92), VirC2 (pdb code 2rh3), DndE (pdb code 4lrv) and AvtR (pdb code 4hv0). First on the left is the simplified representation of the smallest-known trefoil knotted protein (MJ0366 residues 6–92, pdb code 2efv). For each structure of knotted protein are marked the N-terminal knot tail length (red) and the C-terminal knot tail length (blue).

The second structure is a C-terminal domain of VirC2 from Agrobacterium tumefaciens [30] (pdb code: 2rh3), which has the same fold as MJ0336_CC but low sequence similarity (21%) and is longer by 34 amino acids. The third protein is DndE from Escherichia coli [31] (pdb code: 4lrv), with very low sequence similarity to all other studied proteins, and longer than MJ0336_CC by 21 amino acids. The motif found in the DndE structure does not match the RHH motif, and its function has not been recognised, although it may also be a DNA binding protein [9]. These proteins have the left-handed trefoil (−3_1_), which can be characterised by knot core length and so-called tails of the knot (the number of amino acids from C- and N-termini) as determined by KnotProt [7]. The depth of the knots for the investigated proteins and their structures is shown in Fig 1. As an unknotted protein we used AvtR from a hyperthermophilic archaeal lipothrixvirus which has the same fold as MJ0366 and VirC2, and is 99 amino acid long (pdb code: 4hv0).

### Coarse-grained model and simulations method

We use the C*α* coarse-grained structure-based model as presented in detail in [32]. Native amino acid contacts are mimicked by the Gaussian potential [33] with the standard parameters proposed by the SMOG server [34]. All simulations were conducted using Gromacs v4.5.4 with the Gaussian potential as implemented in [34]. A leap-frog stochastic dynamics integrator with an inverse friction constant of 1.0 was used. The time step was equal to 0.0005*τ*.

The trajectories in folding simulations were initiated from previously generated set of unfolded conformations. These conformations were generated in independent long runs at a temperature above *T*_*f*_, and chosen randomly with the imposed condition, that the starting conformation must have *Q* < 0.2. The unfolding simulations and equilibrium runs started from the folded structure.

### Chaperonin cage

**Fig 2:**
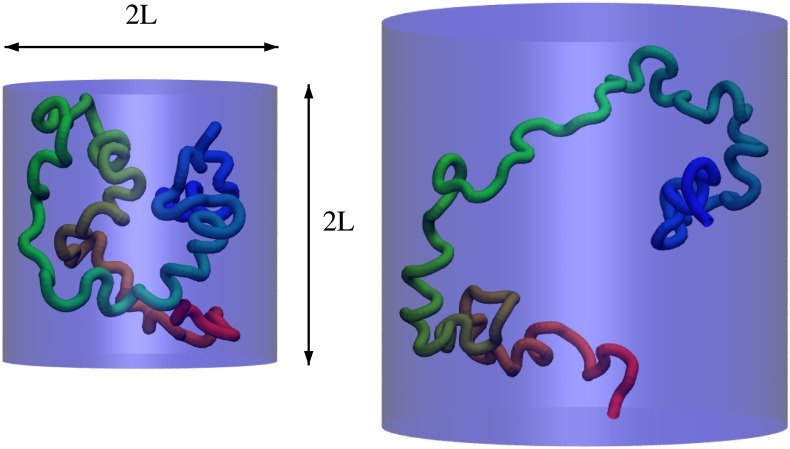
Model of the chaperonin cage, a cylindrical box with a characteristic length L, implemented and applied in this study. Unfolded, unknotted conformations of DndE in a cage with L = 2 nm (left) and L = 3.0 nm (right).

The shape of the cage is cylindrical, with proportions as proposed by Takagi et al. [27], where both height and diameter are equal to 2*L*, hence *L* becomes a characteristic length of the box in which simulated proteins are confined. The chaperonin wall is simulated with the Lennard-Jones potential, $V_{cage}=\epsilon_{c}\left[{\left(\frac{2}{d_{i}}\right)}^{4}-2{\left(\frac{2}{d_{i}}\right)}^{2}\;+1\right]$, where *d*_*i*_ is a distance between the i-th bead and the wall, and *ϵ*_*c*_ = 10.0. The cut-off distance is 2 Å and within that distance from the wall the interaction between each of the beads and the cylinder is present. The geometry of the cage is schematically presented in Fig 2. The wall potential in the form presented above was implemented by us in the version of Gromacs applied in this study.

### Reaction coordinates

To evaluate the progress of folding, the shape of free energy and entanglement we used three reaction coordinates: Q—fraction of native contacts, *R*_*g*_—radius of gyration and topological fingerprint (type of knot, size of the knotted and slipknotted core and its depth), *P*_*K*_(*Q*)—probability of knot existence for a given value of Q. A native contact is considered as formed if the distance between the C*α* atoms is less than 1.2 times their native distance [35].

### Thermodynamics and visualisation

Thermodynamic data were obtained by constant-temperature simulations and histograms at different temperatures were combined using the Weighted Histogram Analysis Method [36]. Structures were visualised using VMD [37].

### Identification of the knot in the protein

The presence of the knot during a simulation was determined in each snapshot using the Koniaris-Muthukumar-Taylor (KMT) algorithm [38] following the procedure described in [39]. Detailed analysis of entanglement was performed based on the topological fingerprint which identified the types of knots formed by the backbone of the entire polypeptide chain and also by all continuous backbone portions of a given protein [40]. To determine the chirality of a knot the HOMFLY-PT polynomial [41, 42] was computed using the Ewing-Millet programme [43] implemented in [40].

## Results

We used molecular dynamics simulations to study how encapsulation influences knotting properties, folding rates and the free energy based on representative set of knotted proteins. We consider proteins with different depth of the knot, sequence and folds, schematically presented in Fig 1. Protein with the deepest knot is MJ0366_CC while the most shallow knot is found in MJ0366 and VirC2. MJ0366_CC has longer the C-terminal tail than MJ0366 by 5 amino acids (the same N-terminal tail), and longer the N-terminal tail than VirC2 by 5 amino acids and the same depth of the N-terminal tail and fold but different sequence. MJ0366 and VirC2 have the knot located respectively closer to the C-terminal and the N-terminal. These proteins share the same RHH fold. The lengths of both tails of DndE are between the shorter and the longest lengths of knot tails of the other proteins, and it has a slightly different fold which is unclassified. We compare the behaviour of these knotted proteins with unknotted protein AvtR from a Hyperthermophilic Archaeal Lipothrixvirus which has the same RHH fold as MJ0366 and VirC2. This set of proteins allows to explore topological frustration based on the depth, location of the knot and its sequence properties. Here we first analyze thermodynamics and kinetics of investigated proteins for different sizes of the accessible volume, and then we identify and characterize corresponding folding pathways.

### Thermodynamics of knotted proteins in the cage versus bulk

**Fig 3:**
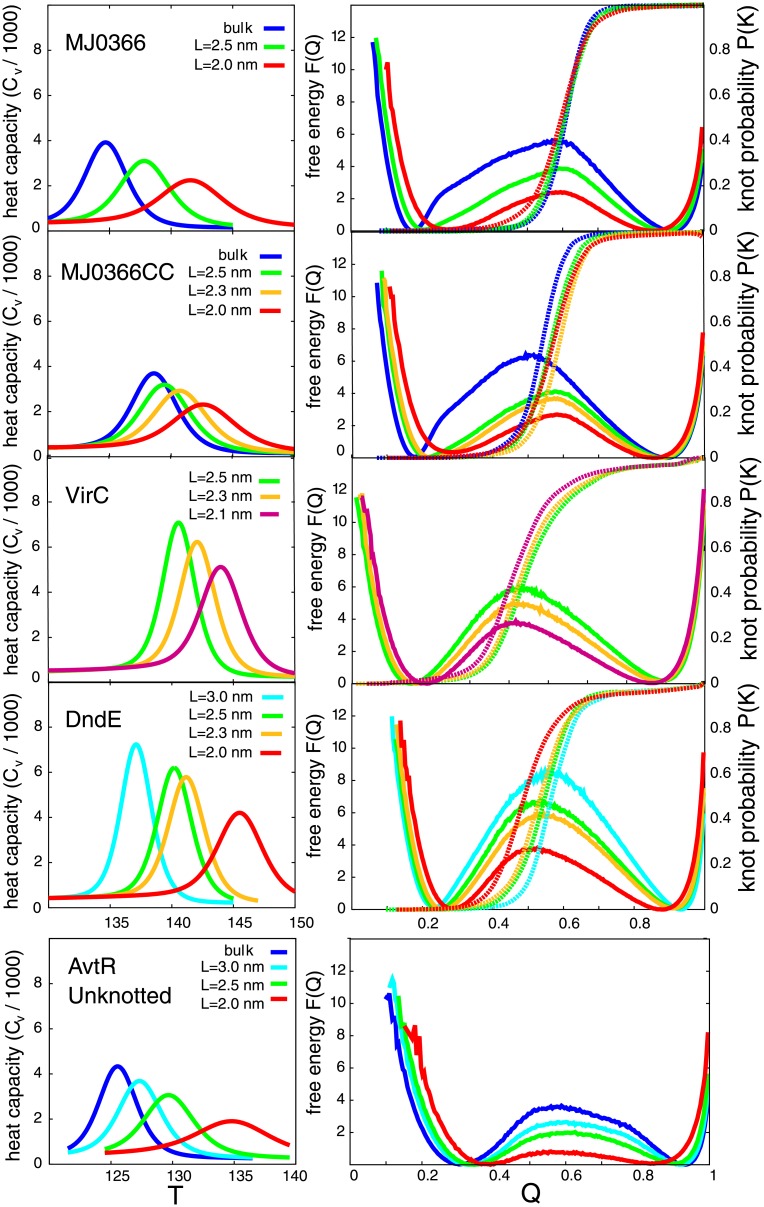
Thermodynamic properties of investigated knotted (MJ0366, MJ0366_CC, DndE, VirC2) and unknotted (AvtR) proteins. Results are calculated at the folding temperature *T*_*f*_, which varies across different confinement sizes. **Left column:** Heat capacity as a function of temperature for several different sizes of the cage, L. **Right column:** Free energy profiles as functions of native contacts fraction *Q* (represented by solid lines) and corresponding knot probability *P*_*K*_(*Q*) (dashed lines) for different confinement sizes.

Our first result is that encapsulation enables reversible folding and unfolding, tying and untying VirC2 and DndE, which is unattainable in bulk. In order to study the thermal stability of the investigated proteins, we determined heat capacity, *C*_*v*_, as a function of temperature for different sizes of the confining cage, based on molecular dynamics simulations. Each capacity curve for the investigated proteins has one peak whose position indicates folding transition temperature *T*_*f*_ as shown in Fig 3. *T*_*f*_ is shifted towards higher temperatures when the cage size decreases as for proteins with trivial topology [27, 29], and thus it implies that confinement has a stabilising effect on knotted proteins as well. Each protein in the bulk solvent has different *T*_*f*_. The lowest *T*_*f*_ was calculated for MJ0366. The heat capacity dependence was calculated using WHAM analysis for an accesible range of temperatures. For DndE and VirC2 at bulk such a method provides only a rough approximation, since we were not able to generate equilibrium runs in these conditions. In our estimations of the *T*_*f*_-s in these two cases, we also based on the results obtained in the confining conditions. Thus, the assed values for *T*_*f*_ for DndE and VirC2 in bulk solvent (134 and 132.5, respectively) must be treated as a rough approximation. The determined value of *T*_*f*_ for MJ0366_CC is therefore considered to be the highest one. At *L* = 2.5 nm, proteins MJ0366_CC, DndE, and VirC2 show similar thermal stability. The largest shift in *T*_*f*_ is observed for MJ0366 and DndE, proteins with a more shallow knot. As we show below, in the case of a more shallow knot, confinement shifts knotting probability closer to the denatured state along *Q*, which results from higher thermal fluctuations and stabilisation due to the repulsive wall.

Moreover, when *T*_*f*_ is shifted to higher temperatures, the peak becomes broader and folding transition is not very well defined. Broader *C*_*v*_ arises partially from more compact non-native structures in the denatured state, as the cage size decreases. As shown below, these compact non-native structures are much more ordered than in the bulk solvent (however, they are not knotted).

The free energy profiles *F*(*Q*) in Fig 3 show that with the decreasing box size, the barrier for the folding-unfolding process becomes lower. The unfolded state minimum is shifted towards higher *Q* with the decreasing cage size, while the native state minimum is slightly shifted in the opposite direction along *Q* for all four investigated proteins. For MJ0366, the free energy curve in the bulk solvent has a characteristic bend close to *Q* = 0.25, which was reported and analysed in previous studies [44]. This bending disappears in profiles obtained for simulations of MJ0366 under confinement.

The thermodynamic data of AvtR, an unknotted protein with the RHH fold, show a similar behaviour. The heat capacity peak shift between unconfined conditions and the cage with L = 2.0 nm is about 10 temperature units, which is similar to the temperature shifts observed for the knotted proteins, based on the estimated value of heat capacity in the bulk. The free energy barrier in the bulk solvent is about 33% lower than for MJ0366 and MJ0366_CC, and in the cage with L = 2.0 nm it almost disappears (more than twice as low as for MJ0366 and MJ0366_CC). It seems that the unknotted protein experiences the confinement much stronger than its knotted counterparts.

Comparison between proteins with the same fold and comparable length shows that knotted proteins are significantly more stable than the unknotted one, both in the cage and bulk. The heat capacity peak is shifted by at least 10 temperature units between two groups. Comparison between the same protein with different knot depths shows that a deeper knot increases protein stability (*C*_*v*_ is shifted by 5 temperature units). Moreover, the data suggests that as the size of encapsulation decreases proteins with a deeper knot (MJ0366_CC, VirC2) gain less stability than those with a more shallow knot (MJ0366, DndE) and the unknotted one (AvtR).

### Knotting is a rate-limiting step even in confinement

To monitor knot formation along the reaction coordinate, we determine the probability of knot existence *P*_*K*_(*Q*) for a given *Q* at *T*_*f*_ for different sizes of cage *L*. Superposition of *P*_*K*_(*Q*) on *F*(*Q*) is presented in Fig 3 by dashed lines. Independently of encapsulation and its size, *P*_*K*_(*Q*) for all proteins has a sharp hyperbolic-tangent shape with its inflection point strongly correlated with the position of the free energy barrier. These results imply that the knotting event is still the rate-limiting step and the cage does not change the general free energy shape, but rather smoothens it as the barrier size decreases. This suggests that the restriction of conformational entropy increases the probability of the protein chain to be in a conformations appropriate to overcome the transition state, without changing the folding and knotting mechanism.

Subsequently, we analyse the position of *P*_*K*_ along Q with respect to the nano-cage size. For all proteins encapsulation in the broader cage shifts *P*_*K*_ to higher Q with respect to unconfined conditions (*P*_*K*_ for VirC2 and DndE was estimated). However, as the size of the nano-cage decreases (and folding temperature increases), *P*_*K*_ is shifted in the opposite direction towards lower Q. The largest shift is observed for the protein with the most shallow knot (DndE). In the case of the deepest knotted protein (MJ0366_CC) a shift towards lower *Q* is observed from L = 2.3 nm; for L = 2.5 nm *P*_*K*_(*Q*) it still moves towards higher Q. In general, weak confinement probably delays (along Q as a coordinate) knotting probability, while tight confinement increases knotting at low Q, at *T*_*f*_ relevant for a particular cage. The cage size at which turnover appears depends on knot depth and local sequence properties of a protein.

In fact, it has been shown that the probability of knotting for polymers or biopolymers such as DNA increases as the volume of encapsulation decreases [45–49] up to some range of compactification. Our results show that the value of *P*_*K*_(*Q*) is almost zero in the vicinity of the unfolded basin (Q<0.3) for all proteins even at the smallest cage (L>2 nm). This shows that no random knots (not in the native position along a sequence) are formed during very early stages of the folding process, contrary to what is expected for polymers. Such knots, when located in the native position, could be used as nucleation sites and lead to a much faster folding, as shown experimentally and theoretically for 1j85 [16, 50]. For moderate cavity sizes the system behaves in a way similar to the case of no confinement. Encapsulation in the smaller box generally shows higher probability of knotting in less ordered structures.

Influence of the smallest cage on the probability of knotting seems to depend on knot depth, as follows from the comparison of data for different proteins. For a very tight cage (with L = 2 nm) a significant number of knotted chains is observed at Q = 0.4 for the protein with the most shallow knot (DndE). Appearance of knots suggests that the knotted chain at Q = 0.4 is not stabilised by the native conformation and has almost random position. Detailed analysis of protein configurations surprisingly shows that the backbone, even at such low Q, is tying in a slipknot geometry from both termini. A abundant number of knots arise at temperature (*T*_*f*_ at which folding/unfolding takes place is significantly higher), which accelerates random kicks, makes protein backbone more flexible and suppresses the strength of native interactions. In consequence the mechanism of protein knotting resembles polymer tying. In this way correct and stable knotting events may occur, and more frequent folding events can provide thermodynamic data for the process unattainable without confinement.

### Kinetics acceleration

**Fig 4:**
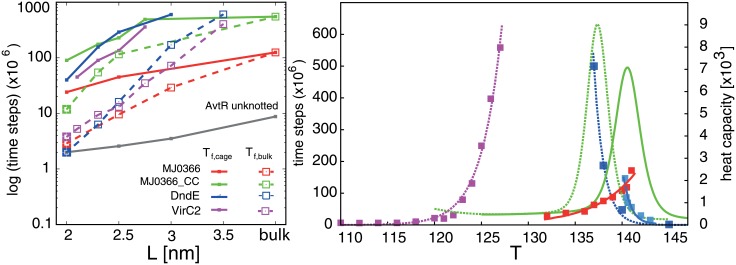
**Left:** Average times between folding-unfolding transitions as a function of chaperonin box size (L) in logarithmic scale. **Right:** Median folding and unfolding times for VirC2 in a bulk solvent (dashed lines, magenta and dark blue squares) and in the chaperonin box with L = 2.5 nm (solid lines, red and light blue squares) as a function of temperature. The heat capacity is presented with green lines (dashed for bulk solvent, and solid for confinement).

We found out that the introduction of restricted space accessible to the investigated proteins significantly affects their kinetics. The average times between folding and unfolding events are presented in Table 1. All simulations were performed at *T*_*f*_ for a given *L*. The protein with the most shallow knot (MJ0366) has the fastest kinetics both in the bulk and in the chaperonin box compared with other knotted proteins, and encapsulation has the smallest impact on its average folding and unfolding time, which is 5 times faster in the chaperonin box with L = 2.0 nm. For the protein with the deepest knot (MJ0366_CC) this factor is around 6. For the protein with the middle depth knot (VirC2) and for DndE this factor is probably much larger (in the bulk solvent we obtained only a few folding events despite simulation times reaching 4 × 10^9^ steps, which provided insufficient data). The average time between transitions as a function of cage size (L) has an exponential behaviour for L between 2.0 nm and 2.75 nm for all the investigated proteins as presented in Fig 4 (Left).

**Table 1:** Average times between folding-unfolding transitions in different confinements for investigated knotted (MJ0366, MJ036_CC, VirC2, DndE), and unknotted (AvtR) proteins. Times are given in simulation steps and divided by 10^6^.

*L*[*nm*]	*MJ*0366	*MJ*0366_*CC*	*VirC*2	*DndE*	*AvtR*
2.0	24.1	90.0	—	40.0	2.0
2.1	—	—	45.0	—	—
2.3	—	175.0	90.0	156.0	—
2.5	45.0	229.0	150.0	291.0	2.6
2.75	—	490.0	359.0	—	—
3.0	—	—	—	602.0	3.5
bulk	125.0	550.0	—	—	8.7

In order to find the kinetic behaviour of VirC2, series of simulations of folding and unfolding processes at different temperatures were performed, in bulk solvent and in a box with *L* = 2.5 nm, see Fig 4 (Right). In the unconfined conditions folding and unfolding times exponentially rise at temperatures separated by 10 temperature units. At low temperatures though, the times needed to reach the native state are very short. Also at higher temperatures the protein denaturates rapidly. The temperature at which unfolding times become very long is just below the heat capacity peak, which also occurs for unfolding under confinement. The relation between the temperatures at which folding times become very slow is strongly affected by the existence of confinement, though. In bulk solvent folding times become very slow at temperatures significantly lower than the temperature related to the heat capacity peak, *T*_*f*_. In the case of confined conditions this temperature is very close to the one of the unfolding process and both curves cross each other at the temperature just below *T*_*f*_. This shows that the influence of confinement is much larger for folding kinetics.

Next, we calculated for the considered knotted proteins the folding times in boxes of different sizes at *T*_*f*_ obtained in unconfined conditions, *T*_*f*,*bulk*_, presented in Fig 4 (Left). For DndE and VirC2, *T*_*f*,*bulk*_ were only assessed. Comparing these results to the times obtained at *T*_*f*_ characteristic for a given box, as presented previously, the kinetics of folding at constant temperature *T*_*f*,*bulk*_ for MJ0366_CC and for MJ0366 is much more dependent on the box size. Folding times rapidly decrease for the box size smaller than 2.5 nm. In the box with L = 2.0 nm MJ0366_CC at *T*_*f*,*bulk*_ folds 10 times faster than at *T*_*f*_ corresponding to this box. On the other hand, even though the folding times for VirC2 and DndE are significantly faster, their changes for confinements with L up to 2.5-2.75 nm are proportional when compared with kinetics in *T*_*f*_ characteristic for a given box. The complexity of results may arise due to a fact, that the proteins differ with size (e.g. VirC2 is almost 40% longer than MJ0366_CC), but also with the depth of the knot, which varies between studied proteins.

The unknotted protein (AvtR) folds significantly faster than the considered knotted proteins (about 10 times faster than the protein with the most shallow knot, MJ0366), which is the fastest folding knotted protein considered in this work. For AvtR we also observe the exponential dependence of box size L on folding times.

### Knotting pathway in a middle-sized cage

Below, we will characterise a general folding mechanism of all the knotted proteins considered in the middle-sized cage, *L* = 2.5 nm. We used average contacts maps *S*_*i*_(*Q*) which describe the nativeness of proteins at different *Q* values, with detailed visual inspection.

**Fig 5:**
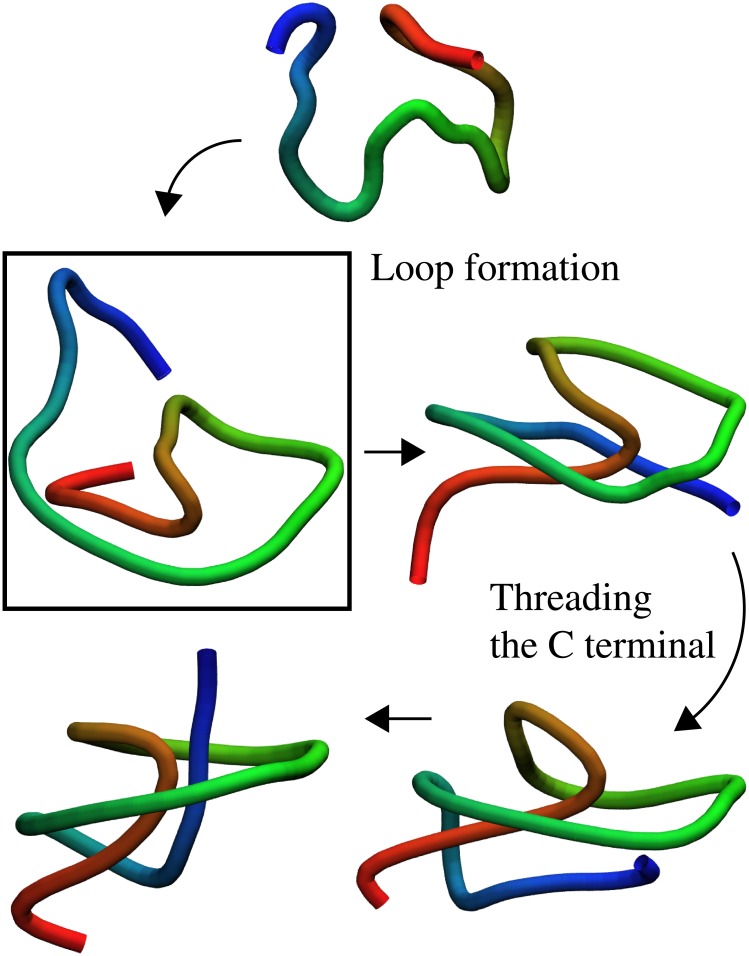
Characteristic folding pathway for MJ0366. The key moment during the folding process is a formation of the *β*-sheet at the N-terminal. The threading of the C-terminal through the loop is the final event of the process.

The sequence of events during the folding of proteins MJ0366, MJ0366_CC and DndE, despite different knot depths and very low sequence similarity (between MJ0366_CC and DndE) is similar, as schematically presented in Fig 5. First, the N-terminal end forms a significant number of contacts with the other fragments of the polypeptide chain. In case of MJ0366 and MJ0366_CC, this leads to the formation of a *β*-sheet, and in the case of DndE, which has a much shorter N-terminal end, it forms contacts with the neighbouring *α*-helix in the native state. In the next step, due to the formation of these contacts, a loop is formed. And finally, the C-terminal end gets through this loop, which leads to the native conformation. During the last event the protein gets knotted. MJ0366 folds in this way in more than 90%, MJ0366_CC in 85%, and DndE in 90% of folding events.

**Fig 6:**
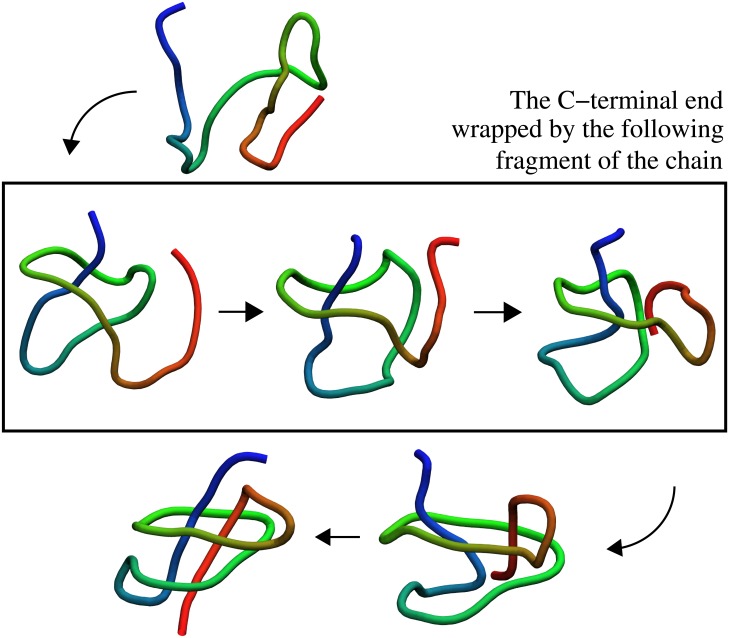
Typical folding pathway for VirC2. First the C-terminal end is wrapped by the consecutive fragment of the chain. In this way the loop is created, and finally the N-terminal is being threaded through it.

VirC2 folds according to the scenario described above only in 10% of folding events. In most cases it folds in the following way, presented in Fig 6. First, the C-terminal helix forms contacts with the fragment of the polypeptide chain which surrounds it in the native structure. During this event, very often both termini come close and arrange parallel to each other. In the last event the N-terminal end makes the last move to knot the protein and to reach the native conformation. VirC2 folds in this way in around 65% of folding events. This scenario of folding is similar, especially in its first stage, to the one reported by Najafi et al. [13]. In this work authors employed a structure-based coarse-grained model in which all information about the native conformation is contained in the optimised angular potential function. In this simplified approach, without introducing interactions between native contacts, it was possible to determine the most populated folding pathway for VirC2 (and for both versions of MJ0366 as well). However, this method does not allow us to observe the complexity of the folding process and competition between different folding mechanisms.

In 25% of folding events with VirC2, the protein reaches its native state in a way in which both termini adopt a native-like arrangement not sequentially, but at the same time. In this case it is not possible to recognise which end makes the final knotting move.

Below, we will compare these pathways to the results for bulk solvent and a smaller nano-cage, based on different methods.

### Comparison of folding pathways in the bulk and in the cage shows that encapsulation causes destabilisation of backtracking

**Fig 7:**
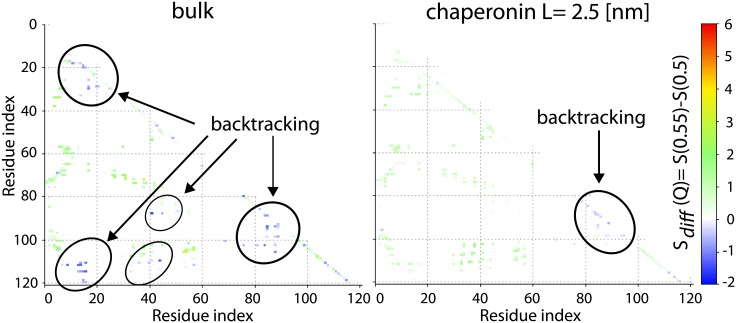
Probability differences of finding formed native contacts for ensambles with Q equal to 0.55 and 0.5 for VirC2 in the bulk and in the chaperonin with L = 2.5 nm. Circled groups of contacts are involved in backtracking.

The role of encapsulation can be partially explained by the investigation of time ordering (along the folding reaction coordinate *Q*) for average contacts maps, in bulk and in the cage. We define *S*_*n*_(*Q*), as the probability of finding the n-th native contact in the cluster of structures with a given *Q*. When no rearrangement between native parts of a protein occurs, *S*_*n*_(*Q*) should get stronger as *Q* increases [51]. Such a scenario is typical for globular proteins with a simple geometry [52]. When part of the protein must unfold before refolding, the difference between the following and the previous contact map *S*_*n*_(*Q*)_*diff*_ = *S*_*n*_(*Q*)_*i*+1_ − *S*_*n*_(*Q*)_*i*_ should be negative. A negative value of *S*_*n*_(*Q*)_*diff*_ defines so-called backtracking [51, 53]. The analysis of *S*_*n*_(*Q*) ordering based on contact maps shows that for all proteins in bulk (for VirC2 kinetics data were investigated) and in the largest cage backtracking occurs around the top of the barrier. However, as the cage size decreases below 3 nm no backtracking is observed. Fig 7 shows examples of backtracking around the barrier height. We found that backtracking appears in similar regions of proteins, i.e. for contacts within *β*-strands and a *β*-strand with a loop. Moreover, the value of *Q* for which backtracking is observed is correlated with knot formation *P*_*K*_ at the top of the barrier. Existence of backtracking implies that when those contacts are too stable, they block the opening of the twisted loop, which is necessary to allow knot tying, or the breaking of *β*-strands to allow threading of the N-terminal end in case of VirC2. In the bulk, the twisted loop does not get destroyed by the folded termini, but in the cage unfolded termini cannot freely fluctuate and thus constantly destabilise the twisted loop. In consequence the loop is less tight and the C-terminus has more space to be threaded through the loop. This suggests that the cage smoothens F(Q) through destabilisation of topological traps and kinetic intermediates on the pathway.

The observed backtracking might be partially a consequence of the fact that with the decreasing cage size, the temperature at which simulations are performed, *T*_*f*_, increases. In order to investigate this dependence, we simulated protein MJ0366_CC at the same temperature, *T*_*f*_, as obtained in bulk, *T*_*f*,*bulk*_, in three different boxes, with *L* equal to 2.5 nm, 2.3 nm and 2.0 nm. The results were compared with those obtained at *T*_*f*_ characteristic for each of the boxes. In the latter case, as it also occurs for all other investigated proteins, the effect of backtracking declines when the box becomes smaller (and temperature increases). In the case of MJ0366_CC simulated at *T*_*f*,*bulk*_, backtracking is present in all three considered boxes. This effect is observed for Q between 0.7 and 0.65, for which *F*(*Q*) has almost the same value (see Fig A in S1 File).

### Folding pathway in the nano-cage is significantly more polarised than in the bulk solvent

Below, we look at how an ensemble of folding pathways is altered by encapsulation. At each Q we identified a set of conformations which belong to the same cluster for a given RMSD. Then, for a given *Q* we compared the size of the clusters in the bulk and in the cage at the same RMSD (from 3 to 6 nm, sampled at 0.25 nm), as shown in 3-dim plots Figs B-D in S1 File. We found that in the cage the largest cluster is significantly more populated than in the bulk up to very high values of *Q*. E.g. for Q = 0.4 and RMSD = 5 nm, the number of structures in the biggest cluser in the bulk and in the chapperon is represented respectively by 9% and 38% of all structures for MJ0366 CC. In the case of VirC2 we compared kinetics data for folding and unfolding routes, see Fig D in S1 File. The much larger cluster size at *Q* = 0.5 (69% versus 18%) and 0.55 (94% versus 37%) in the cage implies that a significant number of chains already has the same residual native-like partial order, even though such *Q* describes rather a disordered structure with native helices. Significantly larger cluster sizes along *Q* imply that protein chains populate the same configuration space, suggesting that the folding pathway is more polarised. If these clusters represent correct structures on the folding pathway, their larger size than in the bulk explains the speed-up in kinetics and smoothening of the free energy landscape.

**Fig 8:**
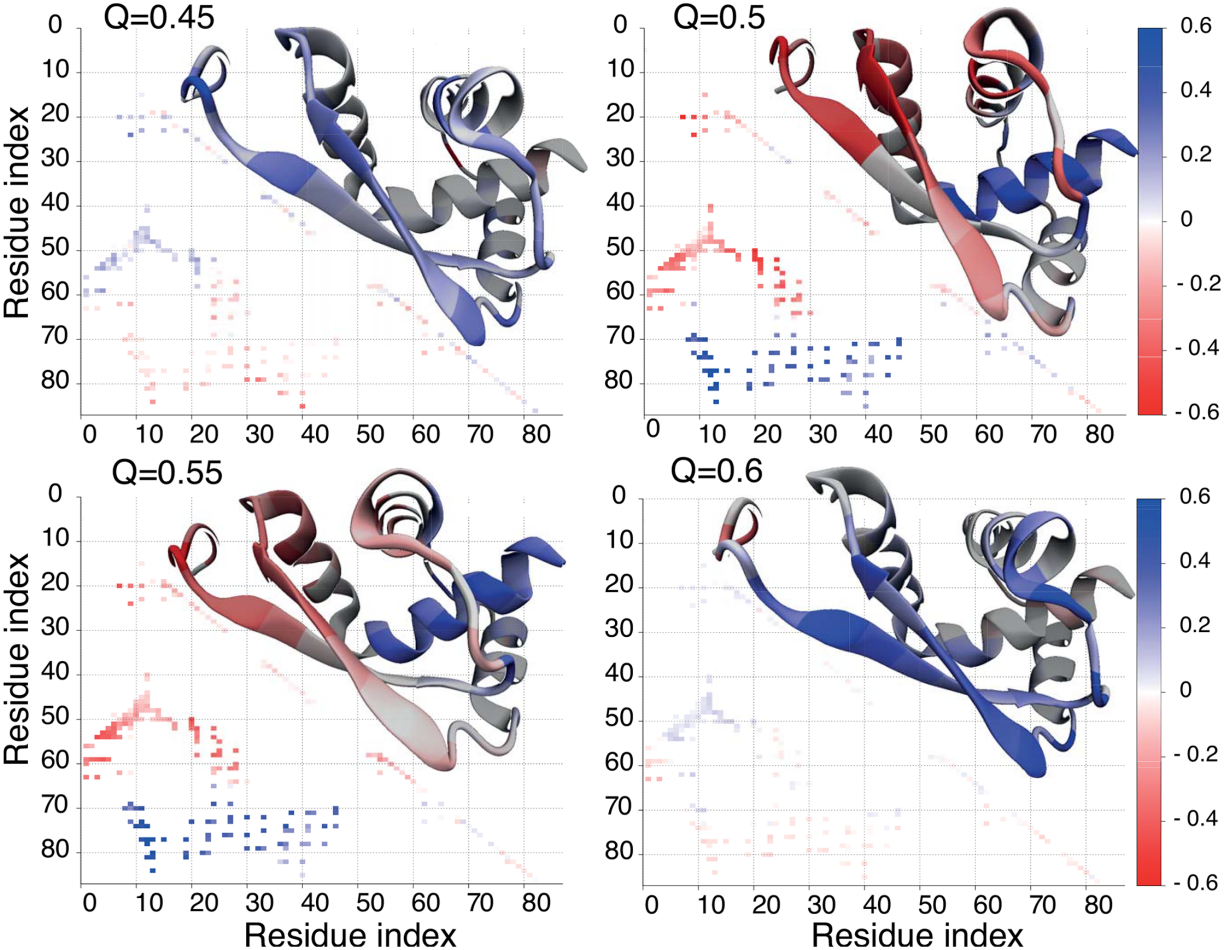
Difference between average contact maps in the bulk and in the chaperonin cage with L = 2.3 nm for MJ0366_CC for selected values of Q: 0.45, 0.5, 0.55, and 0.6. Above the diagonal, the structure of the protein is colored to reflect the changes for each amino acid.

Subsequently, we investigate the geometry of structures inside the clusters and compare it to the average folding pathway in the bulk and in the cage. In the case of MJ0366_CC, the average contact maps in both conditions show similar pattern; however, contacts around the *β*-strands and in the loop are less stable. This folding nucleus is somewhat delocalised and thus the non-native contacts around the twisted loop are more pronounced in the nano-cage. Examples of *P*(*Q*) at *Q* = 0.5 are shown in Fig 8. These differences result in omission of the backtracking step. *P*(*Q*) for the main cluster, with and without the cage, also shows a similar pattern over a broad range of *Q* (it is taken into account that *Q* is shifted by 0.5 between models), data not shown. This suggests that the main folding pathway is not changed upon encapsulation but, on the contrary, it becomes more common.

In case of DndE detailed analysis of folding routes in the bulk and in the middle sized nano-cage (L = 2.5 nm) shows no significant difference. The average contact maps at *Q* = 0.4 and *Q* = 0.5 for different *L* are shown in Fig E in S1 File. A significant difference in the folding pathway takes place at L = 2.0 nm. For such a size *P*_*K*_(*Q*) is around 20% already at *Q* = 0.4, reaching 50% at the top of the barrier at *Q* = 0.47. This implies that knotting is the rate limiting step, but after crossing the barrier the chain has to be rearranged to form native contacts around the knotted chain. Thus in consequence the barrier is closer to the unfolded basin and the native slope of *F*(*Q*) is smaller than in the unfolding basin. An opposite landscape is observed for MJ0366 and MJ0366_CC; *P*_*K*_(*Q*) reaching 50% implies than that the protein chain has almost a native-like shape.

**Fig 9:**
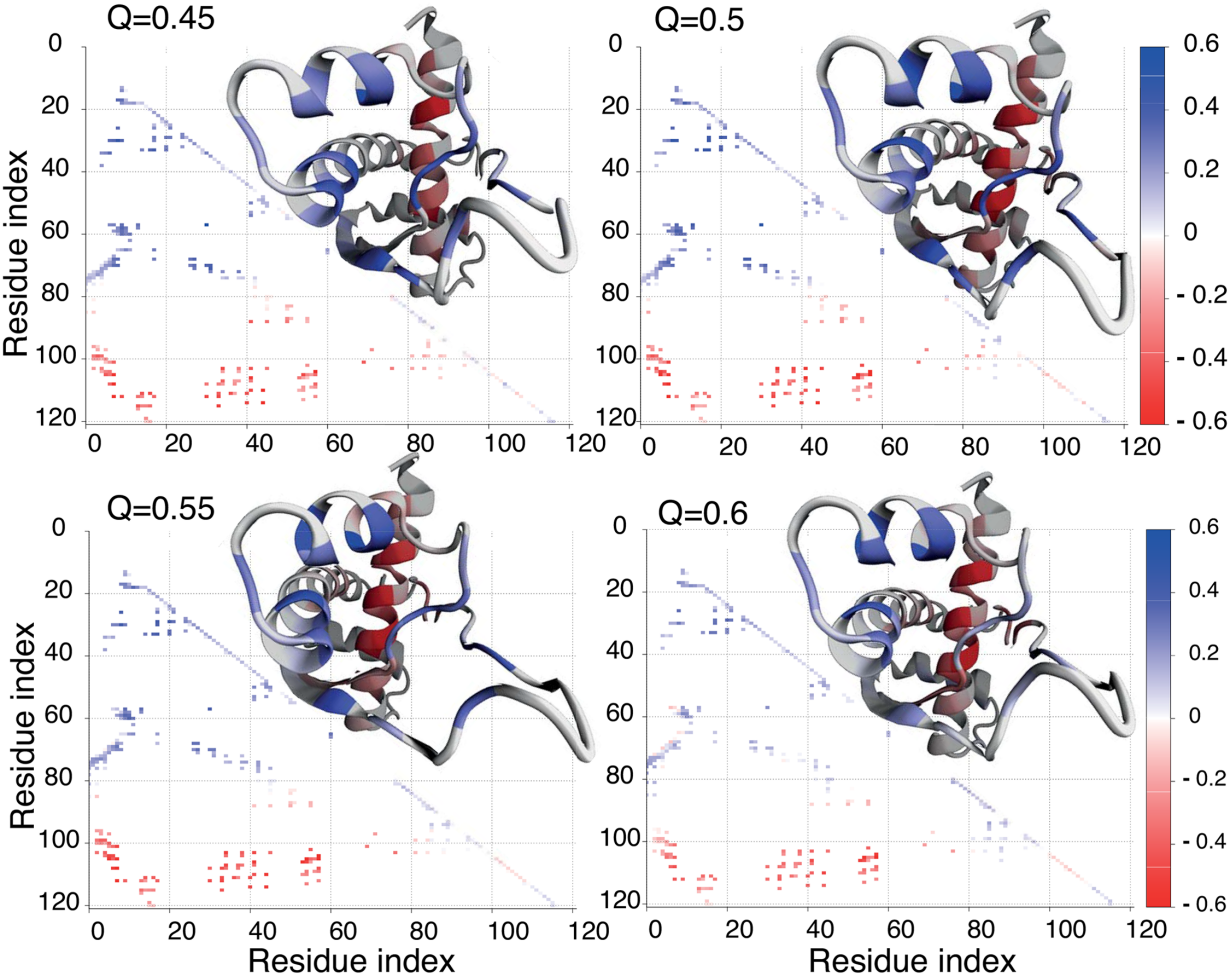
Difference between average contact maps in the bulk and in the chaperonin cage with L = 2.3 nm for MJ0366_CC for selected values of Q: 0.45, 0.5, 0.55, and 0.6. For bulk data was collected at the highest accessible temperature for the folding process in these conditions, T = 127. Data for folding in confinement were collected at T = 140.5. Above the diagonal, the structure of the protein is colored to reflect the changes for each amino acid.

In the case of VirC2 we found that unfolding pathways are similar in the bulk and in the cage. In both conditions the protein unfolds mostly by the N-terminus. However, the folding routes in the bulk and in the cage are significantly different. Fig 9 shows the average contact maps in the bulk and L = 2.1 nm for selected *Q* equal to 0.45, 0.5, 0.55, 0.6, where the difference in the folding mechanism is the most pronounced. In the cage, at *Q* = 0.55 chains are knotted (based on the N-terminal mechanism) or are in the intermediate state where the C-terminus is inside the loop, while at the same *Q* in bulk the structures are unknotted or have an exactly opposite conformation, i.e. the formed *β*-strands and the C-terminal end being outside the loop. The representative structures during folding are shown in Fig 9. The geometries in bulk are similar to those typical for MJ0366_CC and DndE observed here, and those observed for folding with all-atom biased molecular dynamics simulations [14] or other models exactly for VirC2 [11, 54, 55]. The geometry typical for the chaperone pathway in the bulk is observed in less than 10% of folding events. This implies that encapsulation changes the folding pathway for VirC2. However, it has to be taken into account that kinetics data are taken at different temperatures.

### Asphericity

**Fig 10:**
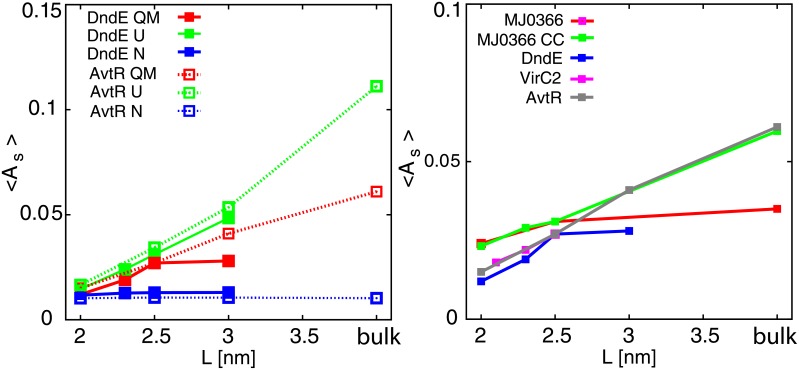
**Left:** Mean asphericity of the native (N), unfolded (U) and at *Q*_*max*_ (QM) ensembles for DndE and AvtR. **Right:** Mean asphericity of the ensemble representing *Q*_*max*_, for which F(Q) reaches maximum, as a function of confinement for the studied proteins.

We applied analysis of asphericity to investigate the influence of confinement on the geometrical shape of the studied proteins. A asphericity is measured as defined in [56]. When its value is 0, it describes a spherical shape, and for asphericity of 1 the object has a rod-like shape. The mean asphericity of the ensemble with *Q* corresponding to the maximum of *F*(*Q*), as a function of confinement size is presented in Fig 10, left panel. The influence of confinement on the geometrical shape of the studied proteins is rather limited. The most affected protein was the unknotted one. The reason for this fact can be that in the case of knotted proteins in structures with *Q* close to the maximum of *F*(*Q*) at least one terminal end is engaged in the threading event and cannot move freely. This restriction does not concern the unknotted protein (AvtR).

For DndE (knotted) and AvtR (unknotted) we analysed the asphericity of unfolded and folded state ensembles and compared them with the results obtained for the ensemble of conformations with *Q* at the maximum of *F*(*Q*), see Fig 10, right panel. The asphericity of the native state is completely unaffected by the confinement of sizes considered in our study. The relatively largest impact of the presence of confinement is observed in the unfolded state ensemble since the restricted space forbids the mostly stretched conformations. These results are consistent with the previous studies of the influence of confinement on small globular unknotted proteins [57].

Additionally, we calculated the radius of gyration for the unfolded and folded states, and for the ensemble of conformations with *Q* corresponding to the maximum of *F*(*Q*), presented in Fig F in S1 File.

The distribution of *R*_*g*_ at the folded state is almost unchanged within the range of considered cage sizes. For an ensemble for *Q*_*max*_, the maxima of *R*_*g*_ distributions are positioned closely together for different confinements, but their widths change with the introduction of tighter confinement.

## Discussion

In this work we examined the influence of confinement on entanglement probability, folding-unfolding rates, knotting pathways and in consequence on the shape of the free energy landscape of four knotted proteins with various depths of knots, sequences and folds. The results are compared to an unknotted protein with a similar fold and length. Due to good agreement with unbiased all-atom explicit solvent simulations [15], we employed a coarse-grained structure-based model of proteins. This choice was supported by the fact that the investigated problem is very demanding computationally and simplifications are necessary to obtain a satisfactory amount of data for a profound analysis of the folding-unfolding kinetics of knotted proteins. The chaperonin-like cage was also introduced in a simplified way, as passive confinement for a protein simulated in its cavity.

We found that the encapsulation of VirC2 (RHH fold) and DndE (unclassified fold) is sufficient to observe the folding and unfolding process of these proteins at equilibrium, which was not accessible in bulk.

The general effect of confinement on the thermodynamics of folding of the considered knotted proteins is similar to that observed for proteins with a trivial topology. With a decreasing space accessible for the simulated protein, its stability increases. However, those results show that the knotted proteins are significantly more stable than the unknotted one, irrispective of the size of encapsulation. As the size of encapsulation decreases, proteins with a deeper knot (MJ0366_CC, VirC2) gain less stability than proteins with a more shallow knot (MJ0366, DndE) and the unknotted one (AvtR). Analysis of entanglement shows that the knotting event is still a rate-limiting step. However, as the size of the nano-cage decreases, a significant number of knotted structures is observed for less ordered structures suggesting that the confined environment of the cell can facilitate knotting of proteins in general.

The free energy profiles calculated for different sizes of the chaperonin-like cage (F(Q)) show that with the decreasing size of the box, the barrier for the folding-unfolding process becomes lower. The impact of confinement does not change the general free energy shape, but rather smoothens it as the barrier size decreases. This suggests that restriction of conformational entropy increases the probability of the protein chain to be in a conformation appropriate to overcome the transition state, without changing the folding and knotting mechanism. This is an important conclusion, particularly for a future study of proteins with deeper knots, for which time scales for the folding-unfolding process at equilibrium in the bulk are computationally inaccessible.

From the kinetic study, we saw that confinement has a significant impact on folding and unfolding rates. For simulations in equilibrium conditions (at *T*_*f*_ characteristic for a given cage size), rates were accelerated up to 5-6 times compared to the results obtained in bulk. With the decreasing cage size, times between folding-unfolding events increased almost in an exponential manner in a range box sizes between 2.0 nm and 2.7 nm for the investigated proteins. For simulations of the protein with the deepest knot (MJ0366_CC) performed in different cages at *T*_*f*_ obtained in bulk, the impact of confinement was even more significant. The folding time in the cage with L = 2.0 nm was 50 times shorter than the folding time obtained in the bulk.

Based on the results obtained for a middle-sized cage we determined the general folding mechanism for the investigated proteins. Proteins MJ0366, MJ0366_CC and DndE share the same sequence of folding events, and threading of the C-terminal end through a loop stabilised by the N-termial end and other parts of the protein to adopt the native knotted conformation is the most common route. In a dominating folding scenario of VirC2, the C-terminal end stabilises a large part of the protein, leading to loop formation, through which the N-terminal end is threaded in the last step to form the native knotted conformation. This striking difference of folding scenarios of very similar proteins might arise from knot depth, and knot tails interactions with other parts of the protein. In the case of MJ0366_CC and DndE, both termini of a knot are almost equal or the N-terminal end of a knot is longer (MJ0366), whereas for VirC2 the C-terminal knot end is almost twice as long as the N-terminal end. The latter is not sufficiently long to stabilise a loop to allow threading of the very long C-terminal end through it. This leads to different scenario of folding in comparison to the other three investigated proteins.

We found that backtracking occurs during the folding process in the bulk in line with the results reported before [11, 12, 15, 44], and when confinement is relatively large. Backtracking is localised around *Q*, which corresponds to the peak of the free energy barrier. However, as the size of the cage decreases below 3 nm no backtracking is observed. The observed backtracking seems to be partially a consequence of the fact that with the decreasing cage size, the temperature at which simulations are performed, *T*_*f*_, increases. For the simulations performed at the same temperature, backtracking was present regardless of the size of the cage.

Clustering analysis confirms that for the studied knotted proteins the main folding pathways remain the same irrespective of whether confinement is present or not. Differences are observed for VirC2 only, which is supposedly a result of the fact that for this protein the folding process is realised on two competing pathways in this kind of analysis.

In this work we investigated the main feature provided by chaperonin presence—the confinement. The further studies of the influence of the confinement on knotted proteins should consider more detailed models of the confining system, with an introduced periodic changes of hydrophobic-hydrophilic changes of wall interactions with a substrate protein, coupled with cavity size changes, to mimic the chaperonin cycles. Possibly, apart of strengthening the pure confinement effect, as it was reported in [25], this can be crucial for the folding mechanism of knotted proteins with deeply located knots.

## Supporting information

S1 File Supporting Figs A to G for the main text. (PDF)
