# Supplementary material for: Knotting and unknotting proteins in the chaperonin cage: Effects of the excluded volume
Source: PLoS One. 2017 May 10;12(5):e0176744. doi: 10.1371/journal.pone.0176744 (PMC5425179; doi:10.1371/journal.pone.0176744)
Supplement: S1 File — (PDF) [file pone.0176744.s001.pdf]

SUPPORTING INFORMATION

Knotting and unknotting proteins in the chaperonin cage: effects of the excluded volume

Szymon Niewieczerzal[1], Joanna I Sulkowska[1,2]

1 Centre of New Technologies, University of Warsaw, Banacha 2c, 02-097 Warsaw, Poland
2 Departament of Chemistry, University of Warsaw, Pasteura 1, 02-093 Warsaw, Poland

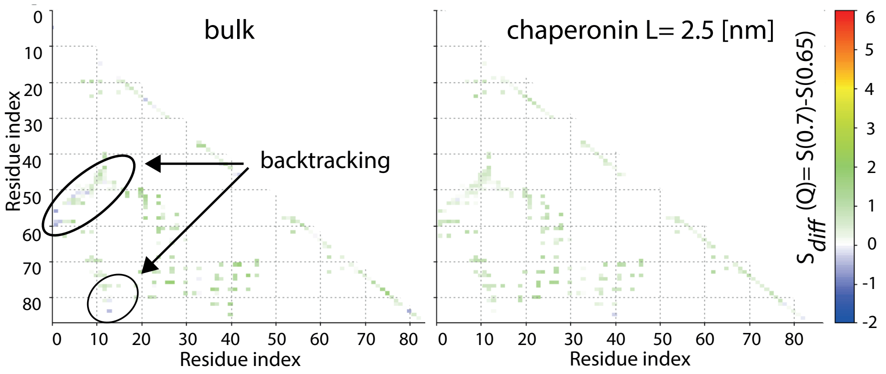

Figure A: Probability differences of finding formed native contacts for ensambles with Q equal to 0.7 and 0.65 for MJ0366_CC in the bulk and in the chaperonin with L=2.5 nm. Circled groups of contacts are involved in backtracking.

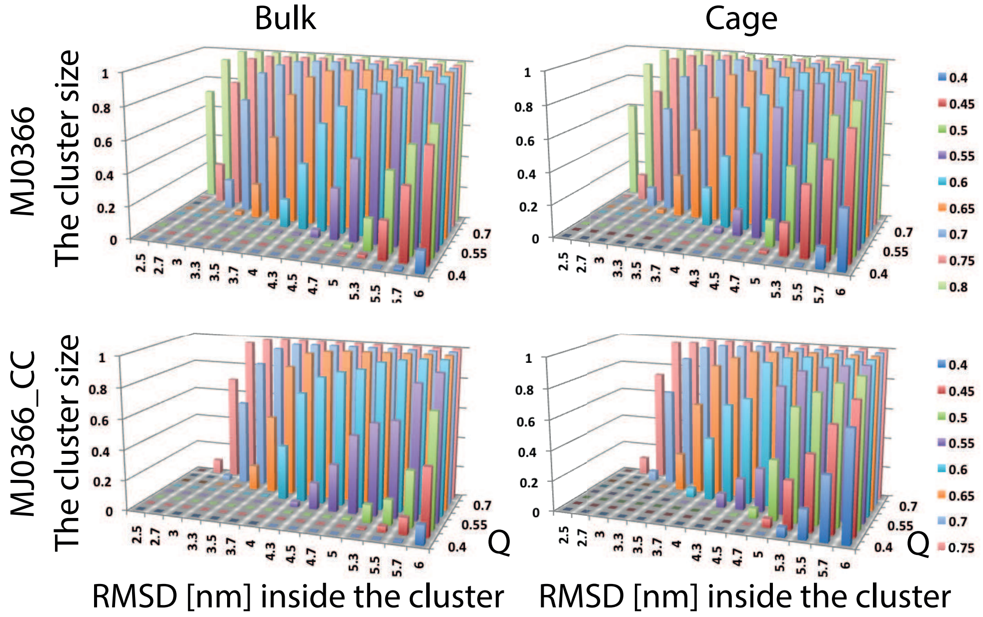

Figure B: Size of the clusters (the number of structures) in the bulk and in the cage at the same RMSD at give Q (0.4-0.8) based on thermodynamics sampling. The top and bottom panel shows data respectively for MJ0366 and MJ0366_CC. The biggest difference is seen around Q=0.45, 0.5, 0.55, 0.65.

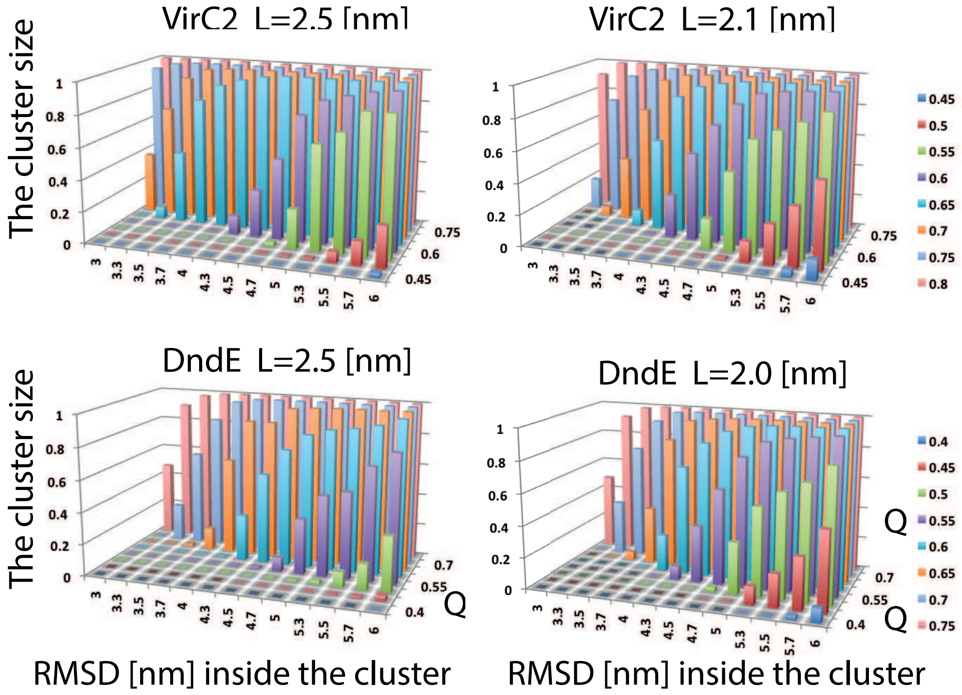

Figure C: Size of the clusters (the number of structures) in bigger and smaller cage at the same RMSD at give Q (0.4-0.8) based on thermodynamics sampling The top and bottom panel shows data respectively for VirC2 and DndE. The biggest difference is seen around Q=0.45, 0.5, 0.55, 0.65.

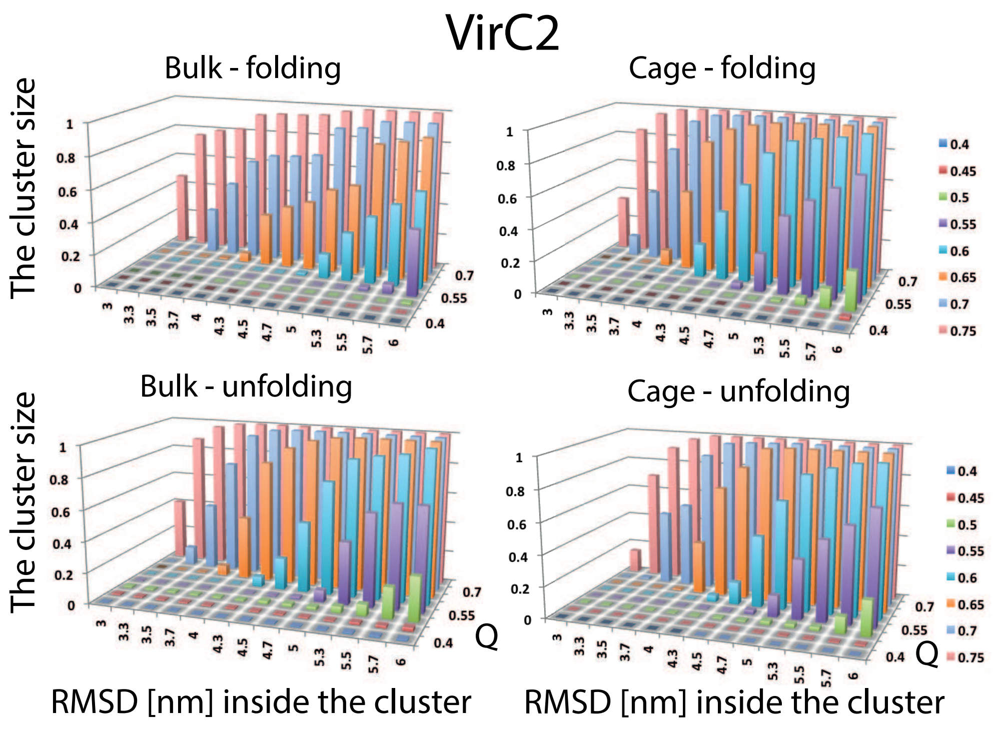

Figure D: Size of the clusters (the number of structures) in the bulk and cage at the same RMSD at give Q based on kinetics sampling. The top and bottom panel shows data respectively for folding and unfolding pathways. The biggest difference is seen around Q=0.45, 0.5, 0.55, 0.65.

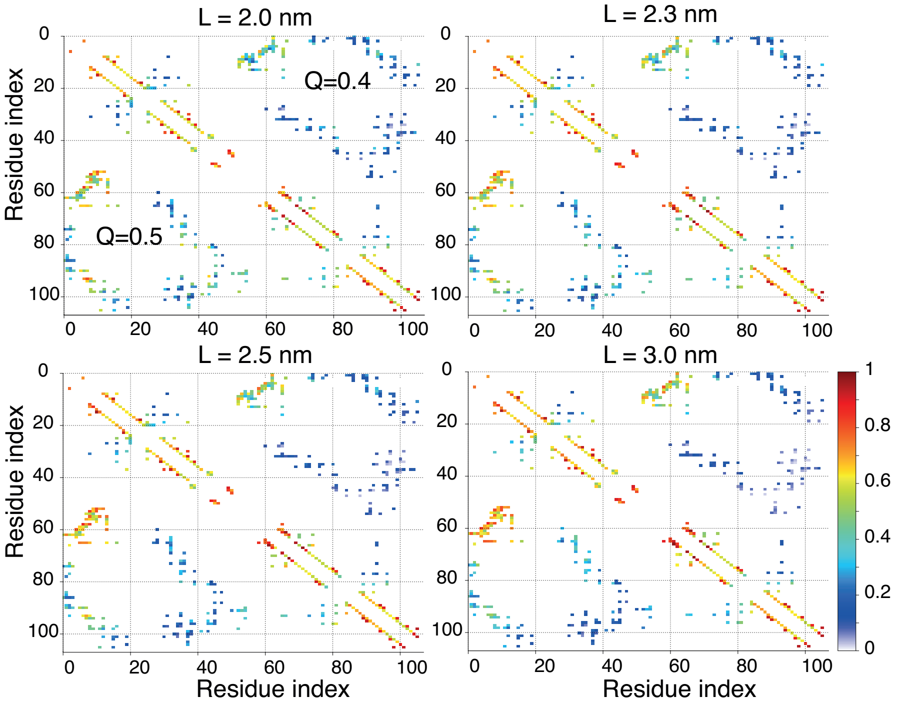

Figure E: Average native contacts maps for protein DndE for Q=0.4 (above the diagonal) and Q=0.5 (below the diagonal), obtained in confining boxes with L equal to 2.0 nm, 2.3 nm, 2.5 nm and 3.0 nm.

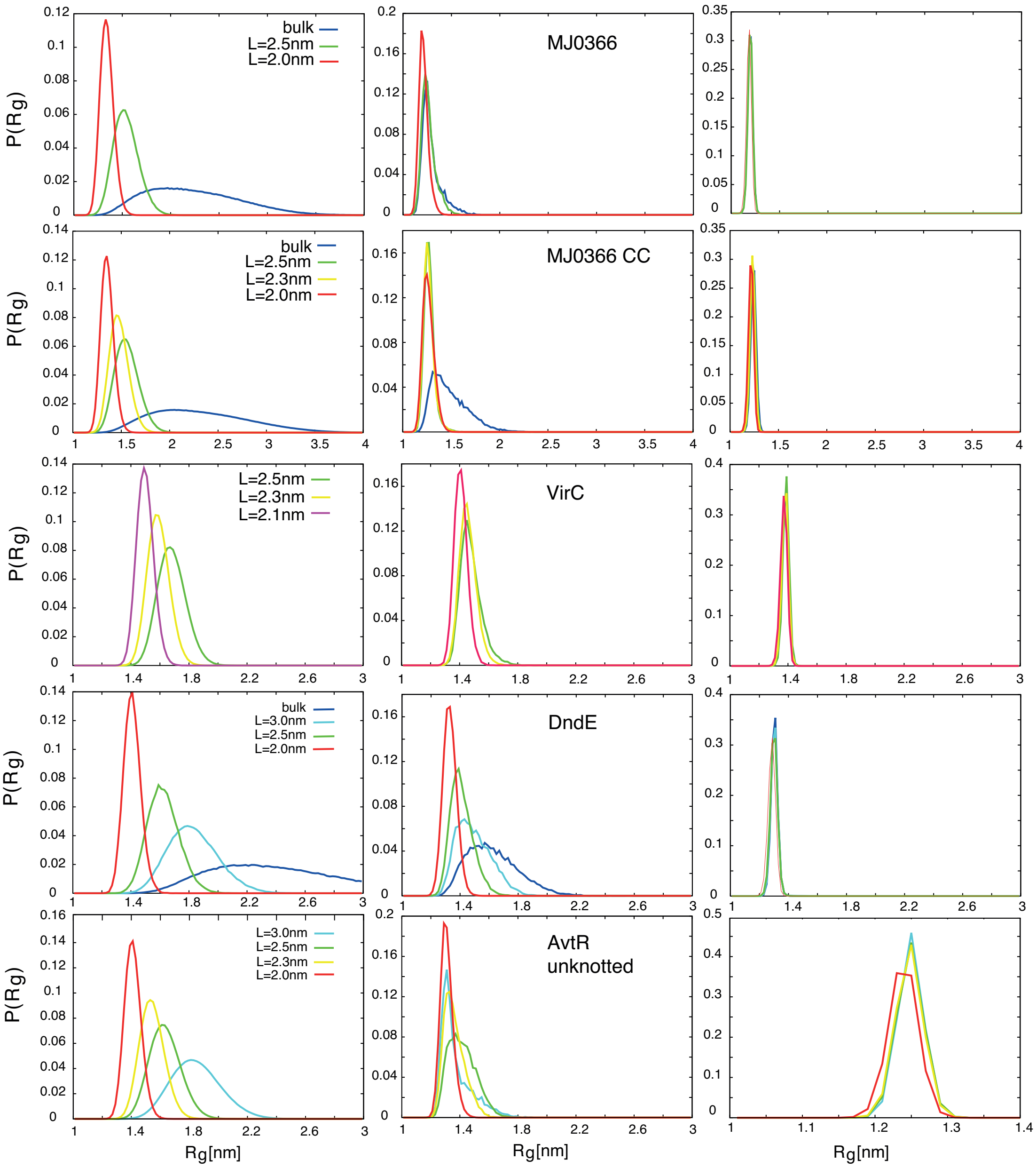

Figure F: Distribution of radius of gyration for the investigated proteins in the denatured state (left column), at Q_{max} (middle column) and in the native state (right column), in bulk and for different box sizes of the confinement. From the top: the knotted proteins MJ0366, MJ0366_CC, VirC2, DndE and the unknotted AvtR, respectively.

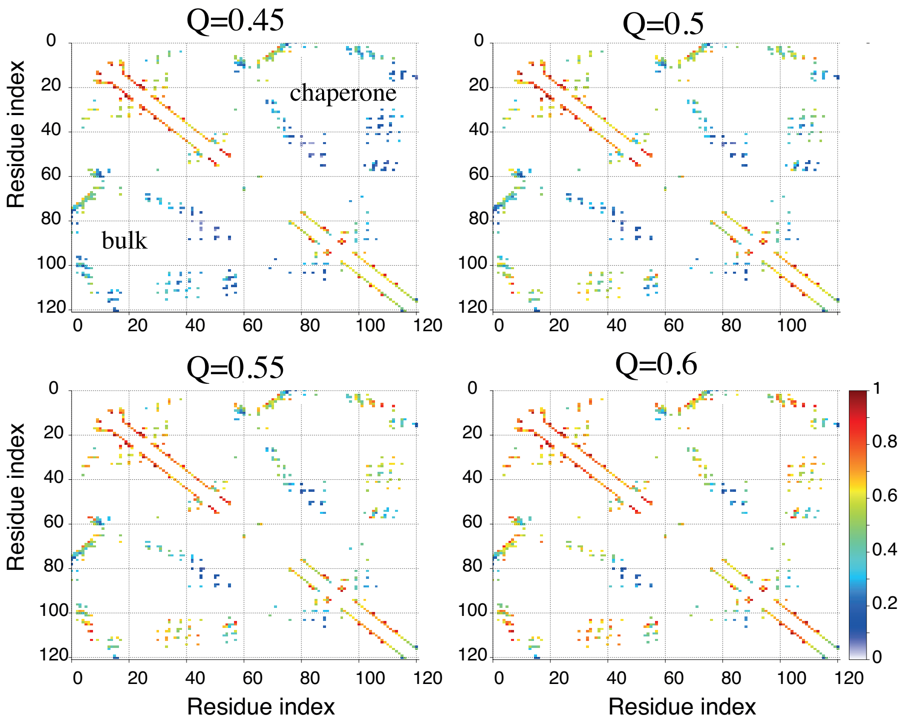

Figure G: Average contacts maps in the bulk and in the nano-cage with L=2.5 nm for selected values of Q: 0.45, 0.5, 0.55, 0.6, for the unfolding process of VirC2. For bulk data was collected at the lowest accessible temperature for the unfolding process, T=137. Data for unfolding in confinement were collected at T=140.5
